# Negative emotional experiences of breastfeeding and the milk ejection reflex: a scoping review

**DOI:** 10.1186/s13006-024-00692-3

**Published:** 2025-03-05

**Authors:** Charlie Middleton, Elaine Lee, Alison McFadden

**Affiliations:** School of Health Sciences, University of Dundee, 11 Airlie Place, Dundee, DD1-4HJ UK

## Abstract

**Background:**

Breastfeeding aversion response (BAR)and dysphoric milk ejection reflex (D-MER) are two examples of breastfeeding phenomena which present as negative emotional experiences of breastfeeding and lactation but are considered physiological in origin. D-MER presents as a sudden onset of dysphoria prior to and during milk ejection. BAR refers to unpleasant feelings and physical sensations during breastfeeding. Research suggests both phenomena are distinct from perinatal mood disorders. As awareness of D-MER and BAR is limited, this scoping review extended beyond the terms D-MER and BAR to describe the nature, extent and range of literature relating to negative emotional experiences of breastfeeding and the milk ejection reflex. The aim of this scoping review was to capture concepts, knowledge and experiences relating to D-MER and BAR.

**Methods:**

This review followed standard scoping review methodology. Medline, CINAHL, MIDIRS, British Nursing Index, PsychInfo, EBSCO, EMBASE, Cochrane Database of Systematic Reviews, Web of Science, Google, Google Scholar, Open Grey, EThOS and PROSPERO were originally searched between March and July 2020 and April 2024, using predetermined keywords. After removing duplicates, records were screened for eligibility. Data were charted, then summarised and synthesised using numerical and thematic analysis.

**Results:**

In total, 116 academic and grey literature records were included in the scoping review and five main themes were identified. D-MER and BAR are associated with a range of negative emotions which impact breastfeeding. Awareness of D-MER and BAR is limited, thus, understanding the phenomena better is important for helping women achieve their personal breastfeeding goals.

**Conclusion:**

This review identified a wide range of literature which described the nature of D-MER and BAR episodes, suggested causes for both phenomena, the impact of D-MER and BAR on women’s lives, strategies women use to cope and possible prevalence rates. Many health professionals remain unaware of D-MER and BAR, and so support provided may be inappropriate or inadequate. Much remains unknown about D-MER and BAR and further research is indicated.

**Supplementary Information:**

The online version contains supplementary material available at 10.1186/s13006-024-00692-3.

## Background

Though breastfeeding is the biological norm for women and infants, barriers to breastfeeding remain common [1]. Societal factors including the marketing of commercial milk formula [2], unrealistic breastfeeding expectations [3] and stigma about breastfeeding in public [4] can undermine infant feeding decisions [5] and perinatal mental health problems sometimes compound breastfeeding challenges [6, 7]. In the last two decades, two previously unheard of breastfeeding phenomena have begun to gain interest; Dysphoric Milk Ejection Reflex (D-MER or DMER) [8–23] and Breastfeeding Aversion Response (BAR) (see also Breast Feeding Aversion and Agitation/BAA) [24–30] now appear in the literature with increasing regularity, but are as yet not widely known or understood.

D-MER is characterised by a sudden onset dysphoria (sadness, anxiety, or anger) which occurs just before and during milk ejection, typically lasting between 30 s and 10 min [9, 16]. Some women describe D-MER as a ‘hollow feeling,’ while others report more extreme emotions including rage and suicidal ideation [8, 9]. Limited research on D-MER suggests it is distinct from post-partum depression or anxiety though it may co-exist with other breastfeeding challenges [9]. BAR manifests as feelings of agitation, disgust, irritability, and physical sensations (such as tingling and skin crawling) which only occur during breastfeeding [25–30]. Although intrusive thoughts are known to affect some women postnatally [31], BAR relates specifically to the breastfeeding act, distinguishing it from perinatal mood disorders [25–30].

The causes of D-MER and BAR are not well understood. Researchers focusing on D-MER appear split into two schools of thought; one suggests the brief drop in dopamine which occurs during milk ejection triggers D-MER in some women [9, 10]. The second suggests D-MER is caused by the release of oxytocin prior to milk ejection [12, 13]. BAR appears to most commonly affect pregnant women, women who breastfeed older infants and toddlers, and women who tandem feed two or more children of different ages) [25]. This has led some authors to suggest that BAR may be an *‘evolutionary mechanism to protect parental resources and increase the chance of further… reproduction’* ([26], p.40).

Knowledge of D-MER and BAR in the maternal health community remains low [8–11, 15–18, 20–30] impacts the support women experiencing BAR and/or D-MER receive [8, 25]. At present, many women self-diagnose with and seek support for D-MER and/or BAR online, yet the adequacy of this approach is not known. This review offers researchers and health professionals the opportunity to better understand D-MER and BAR, and the kind of support which may help women navigate their experiences.

## Methods

### Aim

The aim of this scoping review was to synthesise evidence identified from the literature on D-MER and BAR. To ensure an inclusive approach and to avoid missing literature which described D-MER and BAR using different terminology, this review focused on reports of negative emotional experiences of breastfeeding and the milk ejection reflex.

Specifically, the following question was investigated:

1. What is the nature, extent and range of literature relating to negative emotional experiences of breastfeeding and the milk ejection reflex?

### Design

This review used the scoping review framework described by Arksey and O’Malley [32], a design well suited to identifying evidence on under researched phenomena [33].

### Search methods

This review followed the five stages proposed by Arksey and O’Malley [32] and more recent enhancements of the method suggested by Levac et al., [34], Daudt et al., [35] and guidance from the Joanna Briggs Institute (JBI) [36]:

1. Identifying the research question.
2. Identifying relevant literature.
3. Selecting relevant articles.
4. Charting data.
5. Collating, summarizing, and reporting results.

The inclusion criteria (Table 1) were intentionally wide to avoid missing potential sources of evidence and all types of literature (academic and grey), published at any time, with a specific focus on negative emotional experiences of breastfeeding and the milk ejection reflex were eligible for inclusion. Searches of Medline, CINAHL, MIDIRS, British Nursing Index, PsychInfo, EBSCO, EMBASE, Cochrane Database of Systematic Reviews, Web of Science, Google, and Google Scholar were originally conducted between March and July 2020 as part of a PhD. In April 2024, the search was updated for the purpose of preparing the PhD thesis manuscript. The original search was identical to the updated search. Search terms were developed in consultation with an academic librarian and included; Dysphoric milk ejection reflex; D-MER; DMER; Embod*; Breastf*; Breastf* aversion; Breastf* agitation; Breastf* aversion; Emotion*; Psychological factor*. An example of a search string used is Embod* AND emotional sensation AND breastf*. In addition, citation tracing via reference lists of included records was used to identify additional records which met the inclusion criteria. Due to resource constraints, the review was restricted to studies published in English.

**Table 1:** Inclusion and exclusion criteria

Category	Inclusion criteria	Exclusion criteria
Population	Women of all ages, with experience of lactation in relation to breastfeeding	Women with no experience of lactation in relation to breastfeeding
Concept	Negative emotional experiences of breastfeeding and the milk ejection reflex	Stigma, shame, guilt, pressure (to breastfeed) and practical breastfeeding difficulties. Lack of practical emotional support
Context	All settings considered All published literature relating to negative emotional experiences of breastfeeding	First-hand accounts of experiences of breastfeeding challenges (due to ethical issues of using online data)

Record screening took place in three stages. Stage one involved a review of titles by the Lead Author to identify those eligible for inclusion. In stage two, the lead author and co-authors double reviewed all abstracts independently. Lastly, the lead author and co-authors independently reviewed all full articles and agreed on those included by consensus.

### Data charting

Data relating to record type, author details, publication date, geographical location and content were charted using an adapted data extraction instrument developed by the JBI [21] (Appendix 1).

### Collating, summarising, and reporting results

Records were collated and summarised initially in two data charting tables (one for academic literature and one for grey literature). Subsequently, numerical, and thematic analysis was undertaken. Numerical data relating to the type, geographical origin and publication date of record was presented. In the thematic analysis [21], excerpts of text from included records were examined to identify how the text related to the research question. Academic and grey literature were analysed separately, then codes developed from each group were combined and reported as themes.

## Results

### Search outcome

**Fig. 1:**
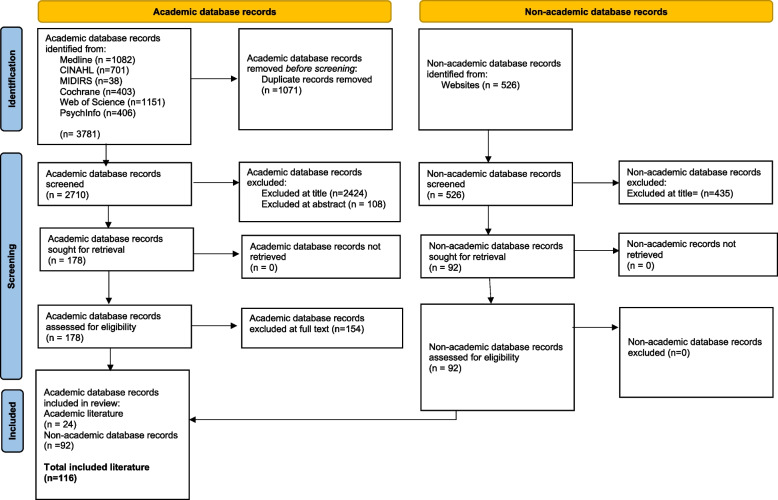
PRISMA 2020 flow diagram for new systematic reviews which included searches of databases, registers and other sources. *Consider, if feasible to do so, reporting the number of records identified from each database or register searched (rather than the total number across all databases/registers). **If automation tools were used, indicate how many records were excluded by a human and how many were excluded by automation tools. From: Page MJ, McKenzie JE, Bossuyt PM, Boutron I, Hoffmann TC, Mulrow CD, et al. The PRISMA 2020 statement: an updated guideline for reporting systematic reviews. BMJ 2021;372:n71. 10.1136/bmj.n71. For more information, visit: http://www.prisma-statement.org/

In total, 3781 academic records were identified. Citations were imported to Endnote and de-duplicated (Fig. 1). In the grey literature search, 526 records were identified (Fig. 1). Citations were inputted manually to an Excel spreadsheet and de-duplicated by hand.

### Findings

One hundred and sixteen records were identified in the search on academic databases (*n* = 24 from academic databases, *n* = 92 from non-academic databases) including two case studies [8, 26], three case reports [9, 19, 21], one case series [10], one letter to an editor [14] and a response to this letter [13)], one commentary [24], one meta-ethnographic review [27], one descriptive study [30], one cross sectional descriptive study [23], one position paper [12], two interpretive phenomenological analyses [12, 29] one qualitative study [25] one mini review [20], one prevalence study [17], one survey [22], one cross sectional survey [28], one integrative literature review [15], one correspondence [16], one retrospective study [11], a mixed-methods study [37] and one perspective piece [18]. Academic records originated from several countries including the United Kingdom (*n* = 2), Ireland, (*n* = 1) the United States of America (USA) (*n* = 7), Australia (*n* = 7), Japan (*n* = 1), the Netherlands (*n* = 1), Egypt (*n* = 2), China (*n* = 1) and Turkey (*n* = 1). One study was developed in collaboration between researchers in the Netherlands and the USA. Sixteen academic records related specifically to D-MER [8–23] and seven to BAR [24–30]. One study focused on maternal experiences of embodied emotional sensations during breastfeeding and included information on both D-MER and BAR [5]. The grey literature consisted of 36 articles, 29 blogs, three news articles, three webpages, four websites, 10 social media posts, two forum posts, two interviews and three books. A total of 41 records related specifically to D-MER and 48 to BAR. Two articles described negative emotional experiences of breastfeeding and milk ejection more generally and one article focused on distressing embodied emotions while breastfeeding. All records were published between 2010 and April 2024. Data extraction tables including details for both academic and grey literature can be found in Appendices 2&3, respectively.

### Themes

Five key themes were identified: negative emotional experiences of breastfeeding and milk ejection, suggested causes, coping strategies, potential impacts, and prevalence.

### Negative emotional experiences of breastfeeding and milk ejection

The literature described a range of negative emotional experiences and physical sensations associated with breastfeeding and milk ejection.

#### Onset

A high proportion of records relating to D-MER and BAR described onset as defining features and key indicators of both phenomena (D-MER, *n* = 67; BAR, *n* = 55). A rapid onset of dysphoria, just prior to and during milk ejection was identified as a defining characteristic of D-MER in 100% (*n* = 67) of D-MER related records [8, 9]. Similarly, all BAR records (100%) described how BAR only occurred in relation to breastfeeding (*n* = 55).

#### Duration

D-MER experiences were described as brief, lasting between 30 s and 10 min [8, 9] whereas BAR feelings (once started) would persist until the infant de-latched [25, 26].

#### Emotional response

Records highlighted how manifestations of D-MER and BAR differed. Heise and Weissinger [9] described D-MER feelings as existing on a ‘spectrum’ of sadness, anxiety, or anger, while women experiencing BAR spoke of feelings of revulsion, disgust, and an overwhelming urge for the baby to de-latch [29, 30] (Table 2.).

**Table 2:** D-MER and BAR experiences

	D-MER	BAR
**Onset**	Just prior to and during milk ejection	Infant latching to the breast
**Duration**	Brief- typically 30 s to ten minutes	Entirety of breastfeeding session
**Emotional response**	Sadness, anxiety, anger, or irritation	Repulsion, disgust, a feeling of not wanting to be touched and an overwhelming urge for the baby to de-latch
**Accompanying physical/visceral sensations**	Nausea, loss of appetite, extreme thirst, hunger, ‘sinking’ feeling	Skin crawling, pins and needles, throat tightening, gut wrenching

#### Physical/visceral sensations

In addition to emotional responses, many women also described physical/visceral responses to D-MER and BAR. For D-MER, these included nipple pain during milk ejection [23, 36], nausea, food revulsion, appetite loss [21] and extreme thirst [37], whereas for BAR, women commonly experienced skin- crawling, tingling, and prickling, throat-tightening and gut-wrenching sensations [24, 25, 29] (Table 2).

### Suggested causes

Several causes for BAR and D-MER were proposed. Ovulation and menstruation were suggested as increasing the likelihood of BAR [25, 26], suggesting hormonal shifts may trigger BAR for some, and many women described BAR as more common when breastfeeding while pregnant, breastfeeding toddlers, and when tandem feeding [24–27]. One participant in the qualitative study by Watkinson, Murray, and Simpson proposed a theory for this [5]:

*“It feels like my body is rejecting…the milk theft…from the…[younger]…child … and that’s being expressed by my body like a physical revulsion.”* ([5], p.58).

Two hypotheses were suggested as causes for D-MER. The first was the abrupt drop in dopamine which occurs just prior to the milk ejection reflex [8] The second suggested oxytocin released during milk ejection triggered the fight or flight response in some women [12, 13].

### Coping strategies

Being aware of and understanding D-MER and BAR as discreet phenomena was crucial for helping women cope with their experiences [8, 9, 24, 26], but in general, knowledge of D-MER and/ or BAR amongst the public and maternal health community was considered low [5, 8, 9, 18–20, 22]. Sharing experiences of D-MER with health professionals could lead to misdiagnoses of postnatal depression or anxiety [5, 18, 37]. In contrast, using the internet to share information and access support was considered invaluable [8, 24, 25, 37]. Being believed and having D-MER experiences validated improved women’s ability to cope with D-MER [37], and in general, self-care was reported to alleviate both D-MER [8, 12] and BAR [25–27]. Sufficient nutrition, hydration, nutritional supplements and sufficient sleep were associated with complete cessation of BAR feelings for some [25–27]. Similarly, sufficient sleep was reported to reduce the frequency of D-MER [8, 12]. Practices such as mindfulness, relaxation and skin- to skin mother-infant contact were reported as helpful in alleviating the intensity of D-MER episodes [12].

### Potential impacts

D-MER and BAR impacted women negatively in several ways; both phenomena were described as unexpected, difficult, isolating and frightening, feelings that were often compounded by the limited awareness of either phenomena:

*“I was scared … No one seemed to understand … [one]… friend… looked at me like I was crazy”* ([26], p.38 -BAR).

In addition to primary experiences of anger during BAR, the nature of episodes led to secondary feelings of guilt and maternal failure for some:

*“…aversion is really horrible*,* it takes away that loving feeling … it makes you want to stop [breastfeeding]… all together…then it makes you feel guilty for feeling like this”* ([25], p. 451-BAR).

Women also described feelings of internal conflict with BAR and D-MER, where wanting to stop and wanting to continue breastfeeding co-existed [10, 25, 37].

*“I wanted to keep on breastfeeding*,* but … I… [also]…didn’t want to…it is a…fight inside me every time”* ([37], p.13-D-MER).

However, the role of D-MER and BAR in prompting breastfeeding cessation was unclear; while some women stopped breastfeeding sooner than planned because of D-MER or BAR [8–10, 14, 19, 28, 37], others were able to continue [11, 24, 37].

### Prevalence

Suggested prevalence of D-MER and BAR was described in five records [*n* = 4 D-MER, 11,17,22,23; *n* = 1 BAR, 28]. For D-MER, rates varied widely from 6% [17]− 28% [23]. The study focusing on prevalence of BAR found 23% of participants were affected by the phenomena [28].

## Discussion

The literature identified in this review suggested D-MER and BAR represent two distinct breastfeeding phenomena which significantly impact some women postnatally but are not well understood or widely recognised. The episodes of sudden and intense dysphoria, and/ or unpleasant physical sensations which characterise D-MER and BAR act as additional stressors during what is often a physically and emotionally demanding time. Many women appear reluctant to openly share experiences of D-MER and BAR, preferring instead to seek support and information online [8, 22, 24, 25, 37]. The reasons for such decisions appear manifold. Historically, underrepresentation of women in medical research [38] has led to gender bias in healthcare provision which means women’s health challenges are not as well understood as those which affect men [38]. Many women report experiencing disbelief, dismissal and condescension when reporting health problems [39]. Thus, women seeking support for sex-based health issues may lack the trust to disclose experiences of D-MER and BAR, especially when little is known about either phenomenon. There is also some justification for women to fear misdiagnosis [18, 39]. D-MER and BAR manifest emotionally, and it is possible that health professionals without knowledge of D-MER and BAR may reach inaccurate conclusions, especially if women have pre-existing mental health conditions and because the distinguishing characteristics of both phenomena are not well known known.

This scoping review identified a wide range of literature related to negative emotional experiences of breastfeeding and milk ejection. A total of 116 records from academic and non-academic databases were identified which met the inclusion criteria for this review. Most literature was grey and lay (*n* = 92), and existed as blogs, online articles or social media posts (*n* = 88) written mainly by women describing personal experiences. Far fewer academic articles [*n* = 24] were identified of which just 19 were empirical or secondary research [5, 8–11, 15–17, 19–23, 25–30, 37]. The sharp increase in literature relating to D-MER and BAR since 2020 is positive and suggests that awareness of D-MER and BAR in online breastfeeding communities and amongst health professionals and researchers is growing, potentially due to the increase in grey literature.

The literature identified gives voice to the experiences of thousands of women across the globe. There are distinct differences between D-MER and BAR, and neither are the same as perinatal mental health issues or general mood fluctuations. While D-MER and BAR appear as relatively benign (though unpleasant) experiences, it is unclear why some women continue breastfeeding, while others do not.

### Implications for future research

Despite the increasing interest in D-MER and BAR in recent years, there remains a limited amount of research undertaken on either phenomenon and further enquiry from a range of perspectives is indicated. Direct physiological research on D-MER and BAR mechanisms will be important for gaining better understanding of their causes. Similarly, the impact of D-MER and BAR on early breastfeeding cessation, and how and why women some women continue to breastfeed despite these challenges is important to understand. Lastly, the proportion of women who experience D-MER and or BAR remains unconfirmed, though a recent study suggests D-MER prevalence might be as high as 28% [25]. The first study aimed at identifying the prevalence of BAR took place in 2024 in which 23% of participants reported BAR experiences [28]. This suggests both D-MER and BAR may be more common than previously considered and further studies which aim to replicate existing findings will be important in elucidating the extent to which both phenomena impact breastfeeding women.

### Implications for practice

Most of the literature included in this review indicated that simple awareness of D-MER and BAR was considered important for helping women continue breastfeeding. Yet despite an increase in academic and grey literature relating to D-MER and BAR, many health professionals who work with breastfeeding women remain unaware of both phenomena. D-MER and BAR manifest as negative emotional responses and there is potential for both experiences to be misinterpreted as post-natal depression or post-natal anxiety [18]. This is supported by a recent study [17] which demonstrated that women with D-MER scored higher for depression on the Edinburgh Postnatal Depression scale when compared with women who did not experience D-MER. Raising awareness may allow health professionals to better support women, and health professionals should be encouraged to listen carefully to women’s accounts of breastfeeding challenges and consider “could this be D-MER/ BAR?” when supporting breastfeeding women. Until further research is published, for women who experience D-MER and BAR, simply being believed and having their experiences validated may reduce isolation and provide valuable support.

### Limitations

There are limitations to this review. Despite the wide-reaching inclusion criteria and search strategy, given the growing interest in D-MER and BAR, it is likely some eligible records were missed. Social media posts about D-MER and BAR are published daily thus it is unlikely all were captured in this scoping review. Most literature originated from English-speaking, high-income countries meaning data mainly derived from these populations. The review was limited to studies published in English due to resource limitations, and it is possible records describing D-MER and BAR in other languages were missed. In recent years, however, academic literature on D-MER has been published in Egypt, China, Japan, and Turkey incorporating the experiences of a wider ethnic and cultural demographic.

## Conclusion

In conclusion, this scoping review described the nature, extent and range of literature relating to negative emotional experiences of breastfeeding and the milk ejection reflex.

A wide range of disparate literature was identified from a variety of sources which described the nature of D-MER and BAR, suggested causes for each, and their impact on women’s lives. Though much remains unknown about D-MER and BAR, both experiences affect some women during the post-natal period and a lack of knowledge amongst lay persons and health professionals can predict a confusing and isolating experience. Considering this evidence, further research is indicated in many areas associated with D-MER and BAR.

## Supplementary Information

Supplementary Material 1. Appendix 1. Data charting table update following review of manuscript.

Supplementary Material 2. Appendix 2. Update following review of manuscript.

Supplementary Material 3. Appendix 3. Grey literature data extraction table Update following manuscript review.

## Data Availability

No datasets were generated or analysed during the current study.
