# Supplementary material for: Negative emotional experiences of breastfeeding and the milk ejection reflex: a scoping review
Source: Int Breastfeed J. 2025 Mar 5;20:13. doi: 10.1186/s13006-024-00692-3 (PMC11881379; doi:10.1186/s13006-024-00692-3)
Supplement: Supplementary file 1 — Supplementary Material 1. Appendix 1. Data charting table update following review of manuscript. [file 13006_2024_692_MOESM1_ESM.docx]

| **Scoping Review Details** | |
| --- | --- |
| Scoping Review title: |  |
| Review objective/s: |  |
| Review question/s: |  |
| **Inclusion/Exclusion Criteria** | |
| Population |  |
| Concept |  |
| Context |  |
| Type of evidence sourced |  |
| **Evidence source Details and Characteristics** | |
| Citation details (e.g. author/s, date, title, journal, volume, issue, pages) |  |
| Country |  |
| Context |  |
| Participant | . |
| **Details/Results extracted from source of evidence**(in relation to the concept of the scoping review) | |
|  |  |

## **Appendix1.**
