# Supplementary material for: Negative emotional experiences of breastfeeding and the milk ejection reflex: a scoping review
Source: Int Breastfeed J. 2025 Mar 5;20:13. doi: 10.1186/s13006-024-00692-3 (PMC11881379; doi:10.1186/s13006-024-00692-3)
Supplement: Supplementary file 2 — Supplementary Material 2. Appendix 2. Update following review of manuscript. [file 13006_2024_692_MOESM2_ESM.docx]

## **Appendix2. Data charting table for academic literature**

|  | Citation details | Year of publication | Country of origin | Intervention / record type | Results/ key findings/ content | Summary/ conclusions |
| --- | --- | --- | --- | --- | --- | --- |
|  | Cox. A case of dysphoric milk ejection reflex (D-MER) | 2010 | Australia | Case study | Feelings of ‘dread, sadness and panic in relation to the milk ejection reflex  Lack of awareness among healthcare providers regarding D-MER. | ‘Dopamine theory’ (Heise, 2008) suggested as cause for D-MER.  Medications which increase dopamine levels may reduce symptoms. |
|  | Heise. Dysphoric milk ejection reflex: A case report. | 2011 | USA | Case report | Lack of concentration and periods of ‘extreme unhappiness’ related to the milk ejection reflex.  Limited information regarding D-MER available. | Dysphoria presents just before or during milk release, and is unrelated to nipple contact.  ‘Dopamine theory’ (Heise, 2008) suggested as cause for D-MER.  D-MER symptoms not caused by lived experience of trauma and can occur after previous normal lactations. |
|  | Heise. An Open  Question: The Mystery of D-MER: What can hormonal research tell Us about dysphoric milk-ejection reflex? | 2018 | USA | Letter to editor | Suggests drop in dopamine as the cause for D-MER.  Acknowledges no direct hormonal research relating to D-MER exists.  Suggests no evidence for psychological cause for D-MER. | ‘Dopamine theory’ (Heise, 2008) suggested as cause for D-MER.  Refutes ‘oxytocin theory ’ (Kendall-Tackett and Uvnäs Moberg, 2018). |
|  | Kendall-Tackett. D-MER—Hypothesis Grounded in Previous Research Dysphoric milk ejection reflex: A case report. | 2018 | USA/ Netherlands | Response to letter to editor | Refutes ‘dopamine theory’.  Acknowledges no direct hormonal research relating to D-MER exists | Suggests ‘oxytocin theory’ as cause of D-MER |
|  | McGuire. ‘Breastfeeding aversion and agitation’, | 2018 | Australia | Case study | Feelings of frustration, anger and resentment when feeding older child and/ or feeding both children simultaneously.  Physical sensations such as ‘skin crawling’ and breastfeeding experienced as ‘unbearable’. | BAA suggested as an evolutionary mechanism associated with (but not restricted to) tandem feeding  Awareness/ recognition of triggers can be helpful. |
|  | Morns. Naturopathic support for nursing aversion associated with tandem breastfeeding. | 2018 | Australia | Commentary | Feelings of aversion only directed at older nursing child when feeding simultaneously. | Some tandem breastfeeding mother experience feelings of breastfeeding aversion directed at the older child.  Simultaneous feeding likely to increase feelings of aversion. |
|  | Morns. Women who experience feelings of aversion while breastfeeding: A meta-ethnographic review. | 2021 | Australia | Meta-ethnographic review | Feelings of aversion during breastfeeding described as overwhelming and may negatively affect a women’s sense of self and impact on the mother-infant relationship. | Some women experience feelings of aversion while breastfeeding which are incongruent with their desire to breastfeed. These feelings may negatively impact breastfeeding duration. |
|  | Pettersson. Experiences and knowledge on Dysphoric Milk Ejection Reflex (D-MER) — A study by means of a mixed method design approach. | 2018 | Netherlands | Mixed- methods study | Results described a range negative physical and emotional symptoms of D-MER, its consequences (stressful breastfeeding, impact on mother-infant bonding, and feelings of guilt and self -stigma, health professionals lack of knowledge and suggested treatments.  Awareness of D-MER aided coping. | Symptoms of D-MER include anxiety, restlessness, nervousness, dread, sadness and depression.  Experiences of D-MER vary in nature, intensity, severity and duration.  D-MER and BAA are different phenomena, but share some similarities in presentation.  Further direct research into D-MER necessary.  Awareness of D-MER important for midwives and other health professionals caring for breastfeeding women. |
|  | Ureno. Dysphoric milk ejection reflex: A case series. | 2018 | USA | Case series | Various negative emotions including dread, stomach ‘churning’ and sadness reported in relation to the milk ejection reflex.  Activities which may trigger D-MER or ease symptoms charted- stress thought to worsen symptoms in all cases | Cases suggest evidence of the presence of D-MER.  Further research needed to better understand the cause, prevalence of D-MER and treatment options. |
|  | Ureno. Dysphoric milk ejection reflex: A descriptive study. | 2019 | USA | Retrospective study | A total of 9% of participants reported D-MER. Respondents described feelings of anxiety, sadness, irritability, panic, agitation, and tearfulness. | First study to quantify a prevalence rate.  The experience of D-MER is different from that of post-partum depression. Future direct research into the cause of D-MER required. |
|  | Uvnäs-Moberg The Mystery of D-MER: What Can Hormonal Research Tell Us About Dysphoric Milk-Ejection Reflex? | 2018 | USA/ Netherlands | Position paper | Hypothesises oxytocin ‘spike’ as cause for D-MER  Refutes ‘dopamine’ theory. | Re-programming and stimulation of the oxytocin response necessary to ease D-MER symptoms.  Mindfulness may help symptoms  Increasing fat and protein in diet may ease symptoms. |
|  | Watkinson. Maternal experiences of embodied emotional sensations during breastfeeding: An Interpretative Phenomenological Analysis. | 2016 | UK | IPA | Three themes identified: ‘The unexpected trigger of intense embodied emotional sensations in relation to breastfeeding, incongruent with view of self’, ‘the importance of fulfilling the mother’s role and the mother- infant relationship’ and ‘making sense of embodied emotional sensations essential to acceptance and coping with experiences’. | Breastfeeding may trigger a range of conflicting cognitions and emotions which impact on how mothers view themselves and relate to their children.  Awareness of emotional breastfeeding experiences and individual nature of difficulties enables health professionals to offer person-centred care. |
|  | Yate. A qualitative study on negative emotions triggered by breastfeeding; Describing the phenomenon of breastfeeding/nursing aversion and agitation in breastfeeding mothers. | 2017 | India, Iran, USA and UK | Qualitative study | Participants describe feelings of aversion and agitation whilst breastfeeding, which vary in form, severity, and duration. The experience is characterised by feelings of anger or rage, a skin crawling sensation and an urge to stop feeding. Participants describe feelings of agitation and irritability whilst the infant is latched. A number of mothers who experience aversion still continue to breastfeed, but have feelings of guilt and shame while also experiencing confusion around those feelings. | BAA is a phenomenon that occurs in some women who breastfeed, whereby breastfeeding triggers negative emotions. The reason women experience it is not clearly known. Research is needed to understand its cause, triggers, and strategies to minimise the experience in breastfeeding mothers. |
|  | Lynn Herr et al., Dysphoric Milk Ejection Reflex in Human Lactation: An Integrative Literature Review. | 2024 | USA | Integrative Literature Review. | Five key themes identified: experiences, sensations, and symptom management; biological underpinnings; influence on maternal role and breastfeeding self-efficacy; support, understanding, and awareness; and reduction and cessation of breastfeeding. | Dysphoric Milk Ejection Reflex is a neurobiological condition characterized by low mood and negative feelings during milk ejection and is linked to psychological distress and unplanned breastfeeding cessation. Future research should prioritise interventions aimed at prevention, symptom control, and raising awareness. |
|  | Heise M, Wiessinger D. Re:" Dysphoric Milk Ejection Reflex: Report of Two Cases and Postulated Mechanisms and Treatment" by Liu et al. | 2023 | USA | Correspondence | Corrects Liu et al., regarding language and suggestion D-MER causes suicidal tendencies | Suggests more care with use of language needed when discussing D-MER experiences and suicide. |
|  | Nguyen et al Dysphoric Milk Ejection Reflex: Characteristics, Risk Factors, and Its Association with Depression Scores and Breastfeeding Self-Efficacy. | 2024 | USA | Prevalence study | N= 201 women completed the survey. Twelve women were classified as likely having D-MER (6%). Symptom resolution primarily occurred within a minute to 5 minutes (58%). EPDS scores differed significantly between those with D-MER and those without. Pre-existing depression or anxiety was not associated with D-MER. | D-MER prevalence may be lower than previously reported. Patients with D-MER appear to have lower breastfeeding self-efficacy and higher depression scores. |
|  | Frawley T, McGuinness D. Dysphoric milk ejection reflex (D‐MER) and its implications for mental health nursing. | 2023 | Ireland | Perspective piece | Describes D-MER characteristics and presentation. | D-MER may have important implications for breastfeeding continuation, differential diagnosis and perinatal mental health. Awareness of D-MER is important for the profession of mental health nursing. |
|  | Liu et al.,. Dysphoric milk ejection reflex: Report of two cases and postulated mechanisms and treatment. | 2023 | China | Case report | Two cases of breastfeeding mothers with D-MER who experienced unpleasant emotions during lactation were reported. One woman weaned her baby prematurely after struggling for 6 months, however the other persisted in breastfeeding until her daughter was 18 months with support. Awareness and knowledge of D-MER are insufficient among the public and health care professionals. | The two cases studies about Chinese women with D-MER enrich the knowledge of D-MER. The literature and published empirical studies about D-MER are scarce and further research on D-MER is warranted. |
|  | Deif et al. Dysphoric milk ejection reflex: the psychoneurobiology of the breastfeeding experience | 2021 | Egypt/Chile | Mini review |  | A call for raising awareness about the complexity of breastfeeding and for the need for mother-centered interventions for the management of D-MER and other postpartum-specific conditions. |
|  | Deif R. Case report: Psychotherapy for enhancing psychological adjustment to dysphoric milk ejection reflex. | 2023 | Egypt | Case report | Firstly, an overview  of D-MER, in relation to attachment, the transition to  motherhood, and sexualization of the female body. Secondly, an overview of the psychotherapeutic journey, and  progress achieved over six months. | Describes the  psychological dimensions of breastfeeding  and physiological aspects of D-MER,  emphasising the need for further research into the psychological facets of different breastfeeding experiences. |
|  | Moriyama Y, Nakao Y, Yamamoto N, Oki T. Dysphoric milk ejection reflex among Japanese mothers: a self-administered survey. | 2024 | Japan | Survey | A total of 15.% of mothers experienced D-MER and had more trouble with breastfeeding than those who did not experience D-MER. Coping strategies included distraction, focusing on the child, and, in some cases, cessation of breastfeeding. Most participants did not consult a health professional about D-MER, believing no one would be likely to understand. | Low awareness of D-MER suggests informing and educating the public is important. Support for women with D-MER may help them cope with their symptoms. |
|  | Morns MA, Steel AE, McIntyre E, Burns E. “It Makes My Skin Crawl”: Women’s experience of breastfeeding aversion response (BAR) | 2022 | Australia | IPA | Four themes were identified: involuntary, strong sensations of aversion in response to the act of breastfeeding, internal conflict and effects on maternal identity, connection between BAR and relationships with others, and reflections on coping with BAR. | BAR is unexpected and difficult for mothers. If support is not available, BAR can have detrimental effects on many aspects of women’s lives. |
|  | Morns et al the prevalence of breastfeeding aversion response in Australia: A national cross‐sectional survey. | 2023 | Australia | Cross sectional survey | One in five women experienced a breastfeeding aversion response.  Limited evidence regarding how to support women who experience breastfeeding aversion response. | Breastfeeding challenges are common, however women who are supported to manage breastfeeding aversion response report a positive overall breastfeeding experience. |
|  | Morns MA, Steel AE, McIntyre E, Burns E. Breastfeeding aversion response (BAR): A descriptive study. | 2023 | Australia | Descriptive study | BAR was more commonly experienced when feeding the first-born child , breastfeeding while pregnant or when tandem feeding. The feelings of aversion were experienced mostly for the duration of the feed. More than half of participants reported BAR caused them to end breastfeeding sessions early. Almost half of participants reported receiving no support from health care professionals for BAR. | More support is needed for women who want to breastfeed while experiencing BAR. New public health policies which promote breastfeeding are needed to help women meet their own breastfeeding goals. |
|  | Kacır A, Karabayir N, Karademir F, et al. Impact of Dysphoric Milk Ejection Reflex on Mental Health. | 2024 | Turkey | Cross sectional descriptive study | A total of 28% of participants reported D-MER . Experiences included tension intolerance , hypersensitivity and nausea. Sleeping or resting, being alone and drinking cold water helped women cope with D-MER. | Raising awareness about D-MER and raising awareness amongst health professionals is important for supporting breastfeeding continuation. |
