# Supplementary material for: Negative emotional experiences of breastfeeding and the milk ejection reflex: a scoping review
Source: Int Breastfeed J. 2025 Mar 5;20:13. doi: 10.1186/s13006-024-00692-3 (PMC11881379; doi:10.1186/s13006-024-00692-3)
Supplement: Supplementary file 3 — Supplementary Material 3. Appendix 3. Grey literature data extraction table Update following manuscript review. [file 13006_2024_692_MOESM3_ESM.docx]

**Appendix3. Grey literature data extraction table**

|  | **Author** | **Date of publication** | **Record type** | **Weblink** | **Title** | **Summary** |
| --- | --- | --- | --- | --- | --- | --- |
|  | Aleksandra | 2019 | Blog | <https://www.karmiuszka.pl/blog/karmiuszka-blog/wszystko-o-baa-breastfeeding-aversion-and-agitation> | Wszystko o BAA - Breastfeeding Aversion and Agitation | Described as a mystery in Poland and ‘very rare’ (equated as less than 20% s not really that rare!). BAA can start with newborns and that some women just experience it once. Makes usual suggestions for support and treatments, but also says ‘diagnosis of child's sensory integration disorders or other child's health problems |
|  | Eglash | 2019 | Article- clinical question/ for CPD? | <https://lacted.org/questions/0159-dysphoric-milk-ejection-reflex/> | Breastfeeding and the  Dysphoric Milk Ejection Reflex | Reports dopamine theory.  Symptoms include: anxiety, agitation, paranoia, fear, sadness, tearfulness, depression and worthlessness  ‘Short lived’ duration.  Reports on finding by Ureno et al. (2019), highlighting demographic and prevalence data. |
|  | Iliades | ND | Blog article | <https://blog.pregistry.com/dysphoric-milk-ejection-reflex-dmer/> | A Sinking Feeling Before  Breastfeeding? It Could Be D-MER | Reports dopamine theory.  Symptoms include: sadness anxiety, ‘sinking feeling’ and hopelessness.  Duration of 30-90 seconds  Awareness of D-MER most helpful, but also activities and foods that increase dopamine and avoiding stress, caffeine, dehydration, and fatigue also seem to help. Bupropion (antidepressant) |
|  | Middleton | 2018 | Article | <https://theconversation.com/dysphoric-milk-ejection-the-real-reason-you-might-feel-sad-when-breastfeeding-103896> | Dysphoric milk ejection: the real reason you might feel sad when breastfeeding | Reports dopamine theory. Cites work by Heise.  Describes D-MER as a spectrum. Some women may experience rage while others become anxious or despondent. Experiences of DMER differ in severity and duration. For some, DMER is mild and resolves in a few weeks. For others, symptoms can last for more than a year.  Just knowing about D-MER helpful. |
|  | Roger | 2018 | Article: Interview with Heise | <https://www.llli.org/what-is-d-mer/> | What is D-MER? | Includes excerpts from the book. States D-MER has a physiological rather than psychological cause. Suggests dopamine theory. |
|  | Anonymous | 2018 | Blog:  Personal account | <http://www.thelocallatch.com/2018/07/how-i-dealt-with-nursing-aversion-and.html> | How I dealt with breastfeeding aversion and what to do when breastfeeding makes you angry | CLC report experiences of BAA. Skin crawling/ nails down blackboard physical sensations and feelings of anger. Tried distraction and EFT tapping, eventually weaned. Problem only occurred with older sibling. Deep breathing and recognising it was BAA helped. |
|  | Bruach | 2018 | Blog | <https://annabrauchlactationsupport.weebly.com/milky-musings-blog/understanding-breastfeedingchestfeeding-aversion> | Understanding breastfeeding/chestfeeding aversion | Symptoms include irritability, anger, rage, a “skin crawling” or creepy crawly feeling, intense urge to run away or to harm their child  Triggered by feeding during pregnancy, tandem feeding, feeding older child, past physical, sexual and/or psychological abuse  Hypersensitivity to stressors  Return of menses  Fatigue, lack of sleep  Malnutrition, vitamin deficiencies, and dehydration  Postpartum mood disorders.  Caused by ‘hormonal shift |
|  | Australian Breastfeeding Association | 2018 | Webpage | <https://www.breastfeeding.asn.au/bfinfo/dysphoric-milk-ejection-reflex-d-mer> | Dysphoric Milk Ejection Reflex (D-MER) | Cites Heise D-MER website and book/ excerpts from both |
|  | Bailey | ND | Social Media | <https://www.pinterest.com/pin/574068283741943763/> | | Personal account / pin and photographs |
|  | Balanced Breastfeeding | 2019 | Article | <https://balancedbreastfeeding.com/tag/dmer/> | DMER Archives - Balanced Breastfeeding | Archive of personal accounts of D-MER |
|  | Becoming super mommy | 2013 | Website | <http://becomingsupermommy.blogspot.com/2013/10/all-about-dmer.html> | All About DMER | Personal account  DR suggested had PPD  Directed by Doula to D-MER website- discusses information from this |
|  | belly belly | 2011 | Forum | <https://www.bellybelly.com.au/forums/breastfeeding-problems-support-135/breastfeeding-agitation-169578/> | Breastfeeding agitation? | Personal account relating to agitation and jittery feelings when feeding. |
|  | Bowen | 2017 | Article | <https://www.romper.com/p/how-to-deal-with-breastfeeding-aversion-according-to-expert-2938531> | How To Deal With Breastfeeding Aversion, According To An Expert | Links to works by Yate and Heise. Suggests BAA and D-MER similar |
|  | Breastfeeding | 2019 | Article | <https://wildlivingmama.com/nursing-aversion-and-agitation-yet-another-breastfeeding-battle/> | Nursing Aversion and Agitation: Yet another breastfeeding battle | “2^nd^ worst BF issue after mastitis” Makes you feel seriously mentally ill” Strong urge to de-latch |
|  | Breastfeeding Basics | ND | Blog | <https://www.breastfeedingbasics.com/badass-breastfeeder/nursing-aversion-episode> | My Nursing Aversion Episode | Personal account  Describes disgust, fear irritation and ‘heebeegeebees’  Doesn’t want to wean- incongruent with desire to BF |
|  | Breathnach | 2019 | Article | <https://www.instyle.com/beauty/health-fitness/dysphoric-milk-ejection-reflex-experience> | This Rare Condition Makes Breastfeeding Emotionally and Physically Painful | Personal account  Cites Heise and refers to “In the 2,500-plus women that I’ve encountered with D-MER, I have not found one single common denominator that connects them,” Macrina Heise says. “I haven’t found anything yet and I wonder if we will. |
|  | Cox | 2019 | Newspaper article | <https://www.dailymail.co.uk/health/article-6898367/Mother-31-reveals-rare-condition-dread-releasing-milk-babies.html> | Mother's rare condition made her dread breastfeeding as she reveals it felt like she was 'killing the family dog' whenever she released milk | Relays a personal account  Symptoms dismissed as PND, feelings of homesickness and dread. Mentions dopamine theory. Awareness key to developing coping strategies. |
|  | Daley | 2019 | Article | <https://www.todaysparent.com/baby/breastfeeding/d-mer-depressed-while-breastfeeding/> | This scary condition makes you super sad while breastfeeding | Relays a personal account:  Suggests dopamine theory. Described as a severe dip in mood lasting 30-60 seconds |
|  | Mathis | 2020 | Blog | <https://www.whattoexpect.com/first-year/breastfeeding/breastfeeding-with-dysphoric-milk-ejection-reflex/> | Breastfeeding made me feel sad and depressed | Relays a personal account  Health professionals hadn’t heard of D-MER  Experienced ‘sense of doom’ and ‘my stomach drops’.  Intense feelings for 1-3 mins but feels’ crummy’ for 15 minutes.  Baby also struggling to latch  Experienced D-MER while feeding each subsequent child |
|  | Dimes | 2010 | Article | <https://www.semanticscholar.org/paper/A-case-of-dysphoric-milk-ejection-reflex-(D-MER).-Cox/b661a48725af9e025878edad28635036fd3a7421> | A case of dysphoric milk ejection reflex (D-MER). | Delete links to Cox article |
|  | Heise | ND | Website | <https://d-mer.org/> | Before the Letdown: Dysphoric Milk Ejection Reflex and the Breastfeeding Mother | See website  Dopamine theory  Describes D-MER symptoms and intensity as “colours” (despondency, anxiety or anger), and intensity (mild, moderate and severe) Mild self resolves in 3 months, moderate by month 9 and severe sometimes lasting more than 1 year.  Triggered by stress caffeine, dehydration). Awareness enough to help coping in mild cases, other women with severe D-MER may need prescribing (what?)  Treatments which increase dopamine prove helpful. |
| Repeat of 69 | Dysphoric Milk ejection Reflex | 2020 | Article | <https://breastfeeding.support/negative-feelings-d-mer-aversion/> | Negative Feelings: D-MER and Aversion | Describes D-MER and BAA. Suggests both Dopamine and Oxytocin theories for D-MER. Skin to skin mindfulness and self-care suggested for D-MER. For BAA, hormonal imbalance suggested as a potential cause. Awareness of both suggested as good support. Nausea and physical symptoms of anaphylaxis considered. |
|  | Ellabellaroo | 2015 | Posting in BF support group | <https://community.babycenter.com/post/a58691236/breastfeeding_agitation_while_pregnant> | Breastfeeding agitation while pregnant | Posting in BF support group regarding BAA while pregnant. Described as ’nails on the blackboard’ feeling’ |
|  | Embracing motherhood | ND | Blog | <https://embracing-motherhood.com/how-nursing-aversion-led-to-the-weaning-of-my-15-month-old/> | How Nursing Aversion Led to the Weaning of my 15 Month Old | Describes BAA symptoms as causing depression. ‘Weird physical sensations : ‘nails on a blackboard’ and toe curling, buzzing/stinging/prickling. Tips for weaning also provided. Cause of guilt. |
|  | Fuller | 2020 | Webinar | <http://californiabreastfeeding.org/event/webinar-behind-the-letdown-an-in-depth-look-at-dysphoric-milk-ejection-reflex-d-mer/> | Webinar: behind the let-down, an in-depth look at D-MER | Na- just note |
|  | Funky Forest | 2017 | Blog | <https://www.funkyforest.com.au/blog/my-second-trimester-on-gentleness-taking-no-shit-breastfeeding-agitation> | My second trimester: On gentleness, kindness, taking no shit and breastfeeding agitation | Describes BAA while pregnant as’ spontaneous eruptions of discomfort, frustration or even anger and an overwhelming urge to de-latch. Self-care and ‘herbs’ suggested as good support with symptom management |
|  | Haseley | ND | Summary of podcast | <https://thebirthhour.com/hyperemesis-gravidarum-dysphoric-milk-ejection-reflex-d-mer/> | Hyperemesis Gravidarum & Dysphoric Milk Ejection Reflex (D-MER) | Says v little about D-MER- just that it occurred. |
|  | HayleyQuinn87 | 2020 | Forum posting | <https://community.babycentre.co.uk/post/a33289380/breastfeeding-aversion> | Breastfeeding Aversion | Describes BF toddler and new feeling of extreme irritation. Asks for support |
|  | Hi Mama | ND | Article | <https://www.hi-mama.co.uk/read/breastfeeding-aversion> | Breastfeeding Aversion | Adds/ describes nothing other than recognising BAA occurs |
|  | Hoffmeister | ND | Blog | <https://www.mother.ly/life/i-had-breastfeed-aversion> | I loved breastfeeding my child—until I didn’t | Describes BF with BAA while pregnant as ‘a sense of panic: a blind rage coupled with the physical need to rip my skin off and exit the situation as soon as possible’. Guilt, shame and sadness as feelings incompatible with the plan to BF until 2 years. Eventually stopped for no particular reason. |
|  | Hohman | 2019 | Article | <https://theeverymom.com/a-condition-called-d-mer-made-me-hate-breastfeeding/> | A Condition Called D-MER Made Me Hate Breastfeeding | Describes feelings of intense irritation for 35-40 mins Nausea, out of body experience. Described as nothing external. Feelings of guilt and shames before understanding finding out about D-MER. This helped. |
|  | indigohj | 2018 | BF support group posting | <https://community.whattoexpect.com/forums/september-2018-babies/topic/breastfeeding-aversion-and-agitation-71158656.html> | Breastfeeding Aversion and Agitation | Posts link to article on BAA |
|  | Jacks | ND | Blog | <https://www.livingandloving.co.za/baby-blog/breastfeeding-aversion-heres-cope-guilt> | Do you have breastfeeding aversion? Here’s how to cope with the guilt | Describes symptoms of BAA and cites Yate. Assumes it causes guilt. Suggests acknowledgement , self-care, hormone ‘checking’ and getting professional support as helpful. |
|  | Jennie | 2017 | Blog | <https://www.edspire.co.uk/year_2017/01/08/ending-feeding-breastfeeding-aversion/> | Ending Feeding: Breastfeeding Aversion | Short blog post describing suddenly hating BF 2.5 year old child. Queries if feelings linked to PTSD (to what?). Skin crawling also described. Some kind of poem included. |
|  | Jo | 2013 | Blog | <http://www.seasidebelle.com/2013/10/overcoming-nursing-aversion.html> | Overcoming nursing aversion | Describes trigger points for BAA feelings. Tiredness, mood and pregnancy hormones described as causes. Suggests timetable feeding as helpful. |
|  | jpiont | 2019 | Blog | <https://raisingmidwesttwins.wordpress.com/2019/04/14/nursing-aversion-agitation-2/> | Nursing Aversion / Agitation | Breastfeeding twins  Describes sudden feelings of hatred and skin crawling. Prickling under armpit. Escribes tips/ support/ diet/ hydration and distraction |
|  | Kate | ND | Article | <https://www.parentingcentral.com.au/nursing-aversion-tandem-feeding/> | Nursing Aversion: Tandem Feeding | Occurred with 2^nd^ child ‘caused to growl into a pillow and scare her daughter (the one she was feeding). Usual support- treatment suggested. |
|  | KellyMom | 2018 | Blog | <https://kellymom.com/bf/concerns/mother/d-mer/> | Depression or other negative emotions upon milk let-down (D-MER) | Briefly describes symptoms of D-MER and cites dopamine theory- includes links to other resources. |
|  | La Leche League | 2020 | Webpage | <https://lllbg.org/tag/breastfeeding-agitation/> | Breastfeeding agitation | Translated from Bulgarian  Describes BAA feelings and differentiates from D-Mer and PND. Suggests hormonal causes and usual symptom managements strategies/ treatments but also ‘twisting’ What is this? |
|  | La Leche League | 2016 | Interview | <https://www.llli.org/what-is-d-mer/> | What is D-MER? | Interview with Heise where she describes her experiences of D-MER, but also on support, how she named D-MER and other information from her website. |
|  | Levine | 2020 | Article | <https://www.whattoexpect.com/first-year/breastfeeding/dysphoric-milk-ejection-reflex/> | If you feel sad or panicky during letdown while breastfeeding, you might have this little known condition. | Provides overview of D-MER (dopamine theory, symptoms, causes support etc). Seems much is from D-MER (Heise) website |
|  | Liesel Teen | 2020 | Blog | <https://www.milkandhoney.jewelry/blog/tag/Dysphoric+Milk+ejection+Reflex> | Macy's Breastfeeding Journey with D-MER | Describes experience of D-MER with feelings of Anxiety, despair and disgust. Stets her lactation consultant didn’t know about D-MER, but joining a Facebook group helped (support). |
|  | Lou | 2019 | Blog | <http://www.theedinburghmum.com/tag/breastfeeding-aversion/> | Breastfeeding: When breastfeeding turns sour | Couldn’t reach site |
|  | Loughney | ND | Article | <https://balancedbreastfeeding.com/nursing-and-anxiety-the-truth-about-d-mer/> | Nursing And Anxiety: The Truth About D-MER | Describes D-MER symptoms as extreme anxiety which ended breastfeeding ‘goal’ for first child- then states 2 years on ‘treatment options ‘ available- but this links to a dead link. States acceptance, mindfulness, visualising (the wave) and talking about D-MER as helpful. |
|  | Madison | 2017 | Article | <https://www.babyinfo.com.au/breastfeeding/breastfeeding-aversion> | Breastfeeding Aversion: Everything You Need To Know | Describes BAA in line with Yates’s suggestions. Describes as different to D-MER and not normally with newborns |
|  | Mama Bean Parenting | ND | Blog | https://www.mothernourishnurture.com/2017/12/navigating-nursing-aversion.html#.YAbbqej7Q2w | Navigating Nursing Aversion I stumbled across this image the other day, which 'spoke to me', shall we say: | Describes experience of BAA symptoms then states caused by sleep deprivation Cites usual mediating factors, i.e. sleep, distraction , diet and hydration etc. |
|  | Mary Sauer | 2016 | Article | <https://www.romper.com/p/i-have-d-mer-this-is-what-its-like-10414> | I Have D-MER & This Is What It's Like | Personal account- cites Heise’s and Weissinger case study work and experience. Started2 weeks PN. Describes nausea and depression dn feelings of self-hatred. |
|  | McPherson | 2019 | Interview with Heise | <https://www.romper.com/p/can-exclusively-pumping-help-d-mer-heres-what-expert-patient-had-to-say-18556111> | Can Exclusively Pumping Help D-MER? Here's What An Expert (& Patient) Had To Say | Cites Heise’s dopamine theory and mentions an earlier rat study. Pumping alone won’t stop D-MER symptoms- for some women BF makes it better. |
|  | Meadows-Fernandez | 2018 | Article | <https://www.lamaze.org/Connecting-the-Dots/when-let-down-brings-you-down-exploring-dysphoric-milk-ejection-reflex-d-mer> | When Let-Down Brings You Down! Exploring Dysphoric Milk Ejection Reflex (D-MER) | New lactation consultant describes finding out about D-MER from reading Heise. Cites dopamine theory. |
|  | Meadows-Fernandez | 2019 | Article | <https://www.instyle.com/beauty/health-fitness/dysphoric-milk-ejection-reflex-experience> | This Rare Condition Makes Breastfeeding Emotionally and Physically Painful | Describes atypical personal experience of D-MER and cites Heise’s work. Talks about difference between D-MER and PND |
|  | Medium | 2017 | Article | <https://medium.com/@dmerorg/talking-to-health-care-professionals-about-dysphoric-milk-ejection-reflex-73a0db518158> | Talking To Health Care Professionals About Dysphoric Milk Ejection Reflex | Describes D-Mer and suggests how women can discuss symptoms with health professionals. Cites wok by Heise and Weissinger, Cox and Watkinson. |
|  | Medium | 2018 | Article | <https://medium.com/@dmerorg/weaning-and-the-d-mer-mother-2453c51e92b6> | Weaning and the D-MER Mother | Describes how D-MER may predict early weaning and suggests women should be empowered/ supported to continue with BF |
|  | Medium | ND | Website | <https://medium.com/@dmerorg/the-familiar-emotional-experience-of-dysphoric-milk-ejection-reflex-c922369db40> | The Familiar Emotional Experience of Dysphoric Milk Ejection Reflex | Describes D-MER feelings as not just anxiety/ sadness etc. but as unique and “hauntingly familiar”. |
|  | Milk and Motherhood | 2017 | Blog | <https://milkandmotherhood.com/2017/01/nursing-aversion-or-wanting-to-scream.html> | Breastfeeding Aversion, or ‘Wanting to Scream, Run and Break Things’ | Describes BAA as intense rage. Suggests labelling helps. Consider causes hormonal as only happen 1 day per month. With a 2 year old child. |
|  | Mom Centre | 2020 | Article | <https://momcenter.com.ph/2020/03/25/hate-breastfeeding-you-might-have-breastfeeding-aversion/> | Hate breastfeeding? You might have breastfeeding aversion | Information describing BAA which seems to be directly lifted from Yate website. |
|  | Moorhead | 2016 | Article | <https://www.thesun.co.uk/living/1540784/a-mum-has-finally-spoken-frankly-about-the-emotional-stress-breastfeeding-aversion-can-cause-so-other-women-know-theyre-not-alone/> | I will not sit quiet' A mum has finally spoken frankly about the emotional stress breastfeeding aversion can cause so other women know they’re not alone | Describes personal experience of BAA ‘heebeejeebees’, frustration, irritability bd distress. Calls for BAA to more widely understood. |
|  | Morns | 2013 | Article | <https://www.babycenter.com.au/thread/194209/breastfeeding-agitation--nursing-aversion---what-helps> | Breastfeeding Agitation / Nursing Aversion - What helps?! | Describes typical BAA symptoms’ Skin crawling and an urge to de-latch’ then advises on strategies including distraction and diet, but not hydration. States a personal theory that tis to do with feeling run down |
|  | Motherlove | ND | Blog | <https://www.motherlove.com/blogs/all/negative-emotions-while-breastfeeding> | Negative emotions while breastfeeding | Describes BAA as if from Yate’s book. Nothing new. |
|  | Mrs Nipple | ND | Blog | <https://www.mrsnipple.com/blog/tag/DMER> | A Mother's journey facing a rare breastfeeding condition | Personal account of D-MER. Describes’ terrible unhappiness and irritability. Also states 35-40 m=of being unpleasant to be around |
|  | Multiple | 2013 | Forum | <https://www.mumsnet.com/Talk/breast_and_bottle_feeding/1929675-Breastfeeding-aversion> | Breastfeeding aversion | Forum posting describes BAA as skin crawling and needing to dig nails into hand as a distraction/ coping strategy-replies link with tandem feeing |
|  | Multiple | 2020 | Webpage | <https://en.wikipedia.org/wiki/Dysphoric_milk_ejection_reflex> | Dysphoric milk ejection reflex | Cites various information on suspected causes, symptoms and management of D-MER. Inaccurate information re not much published after 2015 (link this to academic literature). |
|  | Muza | 2020 | Article | <https://cappa.net/2020/03/30/understanding-dysphoric-milk-ejection-reflex-and-breastfeeding-chestfeeding-aversion-and-agitation/> | Understanding Dysphoric Milk Ejection Reflex and Breastfeeding/Chestfeeding Aversion and Agitation | Cites work of Heise (D-MER) and Yate (BAA). Nothing new. |
|  | Nichole | 2018 | Blog | <https://blog.pregistry.com/breastfeeding-agitation-aversion/> | Breastfeeding Agitation And Aversion | Describes typical BAA symptoms and strategies. Also states boundary setting may be helpful. States BAA more common with latch problems/ difficult feeding early on |
|  | Olena | ND | Article | <https://www.peggyomara.com/2019/09/23/breastfeeding-aversion-and-agitation/> | Breastfeeding Aversion/Agitation | Cites work by Yate and Flower to describe BAA, but also personal accounts too. |
|  | Paclac | 2020 | Article | <https://breastfeeding.support/negative-feelings-d-mer-aversion/> | |  |
|  | Pearson-Glaze | 2020 | Article | <https://www.beautifulbreastfeeding.com/when-nursing-aversion-strikes-and-you-cant-stand-breastfeeding/> | When Nursing Aversion Strikes – And You Can’t Stand Breastfeeding. | BAA when pregnant experienced as like ‘ a large child or someone other than own child was BF’. Feelings of disgust expressed. Uses distraction as a coping strategy. |
|  | Stone | ND | Blog | <https://postpartumprogress.com/for-moms-who-feel-bad-before-breastfeeding-this-may-be-why> | For Moms Who Feel Bad Before Breastfeeding, This May Be Why | Discusses D-MER symptoms (typical, short duration-2 minutes), says not surprising women experience this think they have PND /A. Signposts readers to other blogs, websites etc. Doesn’t make suggestion re treatment. |
|  | Psychotherapy Hellas | ND | Article | <https://coffeecakekids.com/breastfeeding-aversion-what-is-it-and-how-can-you-get-through-it/> | | Describes personal account of BAA (skin crawling etc.) which is linked to periods only lasting one day-(so, hormones). |
|  | Rachel? | ND | Blog | <https://evidence-basedmommy.com/nursing-aversion-when-breastfeeding-isnt-magical/> | Nursing aversion (Or when breastfeeding isn’t magical) | Personal account of BAA while pregnant. Typical symptoms described. Suggests magnesium supplements. Sleep, hydration, diet and self-care. |
|  | Radford | 2019 | Article | <https://evidence-basedmommy.com/nursing-aversion-when-breastfeeding-isnt-magical/> | Nursing aversion (Or when breastfeeding isn’t magical) | Duplicate of 73 |
|  | Rap sarda | 2017 | Blog | <https://runningintriangles.com/tag/dmer/> | A Condition called D-MER: When Breastfeeding Makes You Feel Sad | Describes personal account of D-MER as like a panic attack. Pins and needles and dread. Public health nurse suggested may be D-MER |
|  | Regional Medical Center | 2019 | Blog | <https://www.kidspot.com.au/baby/feeding/breastfeeding/i-wanted-to-rip-my-baby-off-as-soon-as-he-latched/news-story/86731f7d7966cd2cd90ef64ce075aa04> | I wanted to rip my baby off as soon as he latched'. | Describes BAA duration as short lived- typical feelings/ sensations |
|  | Revelant | 2017 | Article | <https://www.thecut.com/2016/07/breastfeeding-depression-dmer-dysphoric-milk-ejection-reflex.html> | The Scary Breast-feeding Disorder People Aren’t Talking About | Short interview with Heise on D-MER- no new information, mentions dopamine theory. |
|  | Revelant | 2016 | News article | <https://www.thebump.com/a/dmer-breastfeeding-anxiety-condition> | The Anxiety I Had While Breastfeeding Is a Real Condition | Personal account which them refers to information provided by Heise. Nothing new. |
|  | Rinkunas | 2016 | Article | <https://rmccares.org/2019/10/03/what-is-nursing-aversion/> | What is Nursing Aversion? | Describes typical BAA symptoms according to Yate- nothing new. |
|  | Rookie Moms | ND | Blog | <https://www.momspresso.com/parenting/the-mommyhood-chronicles/article/my-nursing-aversion-experience-when-breastfeeding-seemed-like-torture> | My Nursing Aversion Experience - when breastfeeding seemed like torture | Personal account- describes typical experiences, craves pain as a distraction |
|  | Salilee | 2019 | Article | <https://chaneensaliee.wordpress.com/2019/12/18/nursing-aversion/> | Breastfeeding aversion and agitation/ nursing aversion | Personal account. Typical description ‘liquid rage’, but about the guilt a and causing weaning. Mention this |
|  | Schreier | 2013 | Article | <https://themamacoach.ca/could-breastfeeding-be-making-you-feel-depressed-d-mer-explained/> | Could breastfeeding be making you feel depressed? D-MER explained | ‘Explains’ D-MER phenomena using Heise’s dopamine theory and other information. |
|  | Schreier | 2019 | Blog | <https://www.romper.com/p/why-does-breastfeeding-make-you-emotional-blame-the-hormones-19494631> | Why Does Breastfeeding Make You Emotional? Blame The Hormones | Relates more broadly to negative emotions relating to BF although D-MER mentioned specifically. Describes different hormonal causes. |
|  | Seaside Belle | 2019 | Article | <https://www.scarymommy.com/d-mer-anxiety-breastfeeding/> | I Had D-MER While Breastfeeding, and Here’s What I Learned | Personal account describing anxiety as a key D-MER symptom. Also mentions experiencing PNA concurrently “ cluster feeds- cluster fuck!”. Cites dopamine theory. |
|  | Stobbe | 2018 | Blog | <https://www.mrsnipple.com/blog/tag/Dysphoric+Milk+Ejection+Reflex> | A mother's journey facing a rare breastfeeding condition | Duplicate of 62 |
|  | Strivelli | 2019 | Blog | <https://theeverymom.com/a-condition-called-d-mer-made-me-hate-breastfeeding/> | A Condition Called D-MER Made Me Hate Breastfeeding | Repeat of 85 in a different publication |
|  | Taylor | 2019 | Article | <https://www.theexaminernews.com/why-do-i-feel-depressed-while-breastfeeding/> | Why Do I Feel Depressed While Breastfeeding? | Psychotherapist describes D-MER as per Heise’s definition/ website information. |
|  | The Every Mom | 2012 | Blog | <https://www.breastfeedingbasics.com/badass-breastfeeder/nursing-aversion-episode> | My Nursing Aversion Episode | Describes typical BAA experience with physical sensations such as ‘stinging, prickling and buzzing and a creepy crawly feeling all over my body’. Continues to wean despite difficulties |
|  | Theuring | 2018 | Forum | <https://www.mothering.com/forum/310-breastfeeding-beyond-infancy/1118209-help-nursing-aversion-breastfeeding-agitation.html> | Pregnant and due in March | Personal account of typical BAA experience in a forum |
|  | Thread Starter | 2019 | Article | <https://www.writermomforhire.com/nursing-aversion/> | Breastfeeding aversion; causes, symptoms and ways to cope | Describes BAA while nursing toddler and newborn ‘hair pulling out’. Typical description and advice |
|  | Ureno | 2019 | Article | <https://www.usuhs.edu/tsnrp/presentation-abstract/describing-phenomenon-dysphoric-milk-ejection-reflex-d-mer> | Describing the Phenomenon of Dysphoric Milk Ejection Reflex (D-MER) | Ureno abstract- not new |
|  | van Kilsdonk | 2019 | Website | <https://balancedbreastfeeding.com/tag/dmer/> | DMER Archives - Balanced Breastfeeding | Personal account describing typical D-MER |
|  | Vong | 2019 | Blog | <https://folks.pillpack.com/when-breastfeeding-feels-like-a-dementor-attack/> | When Breastfeeding Feels Like A Dementor Attack | Personal account which describes D-MER as like ‘finding out someone has died’ |
|  | Taylor | ND | Article | <https://babyology.com.au/baby/feeding/breastfeeding/d-mer-why-sadness-can-sometimes-accompany-breastfeeding/> | D-MER: The chemical reaction to breastfeeding that could be making you feel sad | Describes typical DMER and links to Heise website |
|  | Yate | 2020 | Social Media | <https://www.instagram.com/breastfeedingaversion/?hl=en> | Breast Feeding Aversion | Pictures of BF and Yates book |
|  | Zapata | 2019 | Article | <https://www.parents.com/baby/breastfeeding/d-mer-the-little-known-condition-that-causes-you-to-feel-intense-sadness-before-you-lactate/> | D-MER: The Little Known Condition That Causes You to Feel Intense Sadness Before You Lactate | Personal account of D-MER describing typical symptoms which also links to Heise’s website |

## 
